# Development of an insecticide impregnated polymer wall lining for malaria vector control

**DOI:** 10.1186/1475-2875-13-S1-P80

**Published:** 2014-09-22

**Authors:** Mthokozisi Sibanda, Walter Focke

**Affiliations:** 1 Center for Sustainable Malaria Control, Institute of Applied Materials, University of Pretoria, Pretoria, Gauteng, South Africa

## Background

Dichlorodiphenyltrichloroethane (DDT), pyrethroids, organophosphates and carbamates are insecticides recommended by the World Health Organisation (WHO) for malaria vector control [1]. Long Life Insecticide-Treated Nets (LLIN) and Indoor Residual Spray (IRS) are the flagship interventions recommended by the WHO for malaria control. LLIN employ pyrethroids as active ingredients [2]. IRS is most effective when using DDT, a persistent organic pollutant [3]. Insecticide Treated Wall Lining (ITWL) or Netlon^®^ is a slow release technology that combines the advantages of LLIN and IRS. Current commercial wall linings are produced using labour intensive fabric weaving methods. This study explored the effectiveness of inexpensive monofilament mesh linings produced by extruding insecticide impregnated polyethylene and poly(ethylene-co-vinyl acetate) (EVA) directly into a net format.

## Materials and methods

Polyethylene and EVA were extruded with pyrethroids (deltamethrin and alphacypermethrin) and organophosphates (ca. 0.5 wt.% insecticide) respectively through a dye to form monofilament meshes. Scanning Electron Microscopy (SEM) and Confocal Fluorescence Microscopy (CFM) was used to characterize the presence of insecticide on the surface of the monofilaments. Efficacy testing was done using standard WHO bioassay tube tests.

## Results

For Netlon^®^ to be effective, the insecticides have to be available on the monofilament surface since they are contact poisons. SEM and CFM micrographs show that pyrethroids are present and evenly distributed on the surface of the monofilament mesh. The WHO effectiveness criterion is a mortality exceeding 80% after 24 hours following a 30 minute exposure of *An. arabiensis* mosquitoes to test samples. The pyrethroid impregnated mesh samples achieved 100% mortality in this test even after 24 months of storage. Samples impregnated with organophosphates failed to achieve the WHO criterion. Poor performance of the organophosphate insecticides in the EVA matrix was probably due to the high solubility of these insecticides in this polymer.

## Conclusions

Monofilament polyethylene mesh impregnated with pyrethroid insecticides was successfully produced by a simple direct extrusion technique. It is an inexpensive method for the production of slow-release polymer-based wall linings. Laboratory efficacy tests suggest that this ITWL technology may be a potential substitute for IRS and it may also complement LLIN.
